# AMMI and GGE biplot analysis of genotype by environment interaction and yield stability in early maturing cowpea [*Vigna unguiculata* (L) Walp] landraces in Ethiopia

**DOI:** 10.1002/pei3.10068

**Published:** 2021-12-23

**Authors:** Yirga Kindie, Bulti Tesso, Berhanu Amsalu

**Affiliations:** ^1^ Sekota Dryland Agricultural Research Center Sekota Ethiopia; ^2^ Haramaya University Dire Dawa Ethiopia; ^3^ Melkessa Agricultural Research Center Adema Ethiopia

**Keywords:** cowpea, GE interaction, GGE, grain yield, stability

## Abstract

Cowpea is one of the most important grain legumes for human consumption and animal feeding. Despite this importance, its production is hampered by biotic and abiotic constraints. Genotype by environment interaction study was performed to identify the most stable cowpea genotype(s) and the desirable environment(s) for cowpea research in Ethiopia. Twenty‐four cowpea landraces and one standard check were evaluated for grain yield and yield‐related traits at six locations (Sekota, Kobo, Sirinka, Melkassa, Mieso, and Babile) using 5 × 5 triple lattice during 2019. Combined analysis of variance showed that grain yield was significantly affected by environments, genotypes, and GE interactions. AMMI analysis revealed the contribution of environment, genotype, and GEI for 29.79%, 15.6%, and 42.06% of variation on grain yield. The first two principal components explained 57.97% of the total GEI variance. AMMI model selected G24 as 1st and 2nd best genotype at five environments. The polygon view of the GGE biplot identified three mega‐environments (ME1, ME2, and ME3) with winning genotypes: G24, G3, and G16, respectively. The highest productive (2528.8 kg ha^−1^) environment, miesso has been identified as the most; discriminating and representative testing environment whereas the lowest productive (1676.1 kg ha^−1^) Sirinka was the least discriminating and representative. The highest yielder G24 (2632 kg ha^−1^) was identified as the “ideal” and the most stable genotype followed by G16 (2290 kg ha^−1^) while the least stable and low yielder was G11. Therefore, genotypes G24 and G16 were recommended for verification and commercial production in most cowpea growing areas of Ethiopia.

## 1 INTRODUCTION

Cowpea is an annual herbaceous and self‐pollinated grain legume crop that belongs to the family Fabaceae and genus Vigna. It is a diploid species with 2*n* = 2*x* = 22 chromosomes (Ogbemudia et al., 2010). Cowpea is drought resistant and adapted to marginal soil due to nitrogen‐fixing ability that makes it, a useful staple crop for farmers in harsh environments under moisture stress and hot temperatures (Hill, 2015). Cowpea is one of the widely cultivated and consumed grain legumes globally, especially in the arid and semi‐arid tropics (Baidoo & Mochiah, 2014). The crop is cultivated primarily for its edible seeds, young leaves (as a vegetable) and pods, soil improvement, weed management, and soil erosion reduction, and forage value (Mulugeta et al., 2017).

Ethiopia has a great diversity of cowpea landraces (local farmers’ varieties) which vary in many traits and has a high potential for the production of the crop but its production, productivity, and utilization is very low as compared to other African countries though the country is claimed to be center of diversity and/or origin (Mulugeta et al., 2017; Sisay, 2015). This low and unstable productivity across environments and years is due to biotic factors, abiotic factors, and scarcity of widely adaptable and early maturing varieties (Horn et al., 2015). It is known that yield and other traits are influenced by genotypic, environmental, and interaction effects. The variability in environments such as location effect, seasonal fluctuation, and their interaction highly influence the production of cowpea (Adewale et al., 2011). Adeigbe et al. (2011) reported the sensitivity of cowpea for environmental and geographical conditions. Additive main effect and multiplicative interaction (AMMI) and GGE biplot analysis are the best approaches to quantify genotype by environment interaction, classification of mega‐environments and characterization of testing environments, and simultaneous selection of genotypes based on stability and mean yield (Gauch & Zobel, 1996; Yan et al., 2002).

Even though there are many opportunities for breeders to develop cowpea varieties possessing different agronomic characteristics and tolerance to a wide range of biotic and abiotic factors, the progress of cowpea breeding in Ethiopia is very slow, either in exploiting the available genetic variability in the country or from the introduction of improved varieties. Moreover, information on the effect of genotype, environment, and their interaction, and the performance stability of cowpea landraces is scanty. Therefore, this study was initiated to estimate the effects of genotype, environment, and their interaction on grain yield and related traits and to assess the stability of cowpea landrace for yield across environments.

## 2 MATERIALS AND METHODS

### 2.1 Description of the study area

The experiment was conducted in six cowpea growing environments during the 2019 main cropping season. These six locations representing different agro‐ecologies of cowpea growing areas in Ethiopia were selected based on representativeness for cowpea producing environments. Descriptions of these six areas are presented in Table 1 below.

**TABLE 1:** Description of test environments

Environments	Soil type	Altitude (m a.s.l.)	Average rainfall (mm)	Temperature (°C)		Geographical location	
				Min	Max	Latitude (N)	Longitude (E)
Sekota	Vertisol	1850	789	12.9	32.9	12°14′	38°30′
Kobo	Vertisol	1450	673.4	13	34	12°8′	39°18′
Sirinka	Vertisol	1880	876	13.6	27.3	11°08′	39°28′
Melkassa	Andosol	1500	763	14	24.8	8°30′	39°21′
Miesso	Vertisol	1332	787	14.9	28.2	9°28′	38°08′
Babile	Spodosols	1650	671	15.5	28.1	9°30′	42°21′

### 2.2 Experimental materials and design

Twenty‐four Ethiopian cowpea landraces (NLLP_CPC_07‐47, NLL_CPC_07‐74, NLLP_CPC_07‐168, NLLP_CPC_07‐27, NLLP_CPC_07‐05, NLLP_CPC_07‐01, NLL_CPC_07‐49, NLLP_CPC_07‐77, NLL_CPC_07‐46A, ACC 211557, ACC222890, Dass 002, Dass 007, NLLP_CPC_07‐69, NLLP_CPC_07_, ACC 233403, NLLP_CPC_07‐28, NLLP_CPC_07‐46B, ACC 244804, NLLP_CPC_07‐29, ACC 223402, NLLP_CPC_07_48B, NLLP_CPC_07‐03, and NLLP_CPC 07‐55) collected from different regions were evaluated along with one standard check (released variety: Kanketi). The experiment was conducted using 5 × 5 triple lattice design at each location and each genotype was assigned randomly. The seeds were planted on 4 m × 2.4 m (9.6 m^2^) plots having four rows, with inter and Intra row spacing of 60 and 20 cm, respectively. The net harvest area was 4.8 m^2^ per plot, the central two rows. The spacing between plots and blocks was 1 and 1.5 m, respectively. Sowing was carried out in July and all agronomic management practices were done equally and properly as per local recommendations.

### 2.3 Data collection and data analysis

Data for plant height, number of pods per plant, and number of seeds per pod were collected based on five sample plants which were randomly taken from the two central rows and the average of five samples was used for analysis. While, days to 50% flowering, days to 75% maturity, grain yield, and 100 seed weight was collected based on the net plot.

The combined analysis of variance across the environment was done using a mixed model (genotype fixed, location random) using SAS (2009) statistical software package to determine the differences between genotypes across environments, among environments, and their interaction. Mean comparison using Duncan's Multiple Range Test (DMRT) was performed to explain the significant differences among means of genotypes and environments. Additive main effect and multiplicative interaction (AMMI) and GGE biplot analysis were analyzed using GenStat (18th edition, 2015) to quantify genotype by environment interaction, classification of mega‐environments, and characterization of testing environments, and for simultaneous selection of genotypes based on stability and mean yield.

## 3 RESULTS AND DISCUSSIONS

### 3.1 Analysis of variance and estimation of variance components

Bartlett's test indicated homogenous error variance for the grain yield and allowed pooled analysis across environments. The combined analysis of variance of grain yield (kg/ha) and yield‐related traits of 25 landraces tested in six locations is presented in Tables 2 and 3. The analysis showed that cowpea grain yield was significantly (*p* ≤ .01) affected by environment, genotype, and genotype by environment interaction. In agreement with this finding, Santos et al. (2015) and Ishiyaku et al. (2017) reported significant effects of genotype, environment, and genotype by environment interaction on cowpea grain yield and yield‐related traits. The significance of GEI indicated that the relative performances of the genotypes were not consistent across the test environments and the environments had different effects on the yield potential of the genotypes. This, in turn, suggested the need to conduct further analysis on genotype by environment interaction to understand the nature of the interaction, and to identify stable genotypes.

**TABLE 2:** Combined ANOVA for grain yield (kg ha^−1^) of 25 cowpea genotypes tested at six environments

Source of variation	Degrees of freedom	Sum of square	Mean square	Total variation explained (%)
Genotype	24	26479319.29	1103304.97**	20.9
Environment	5	34726016.96	8681504.24**	27.45
Rep (Env.)	12	2615788.67	261578.87**	2.07
Interaction	120	62679547.1	652911.95**	49.55
Pooled error	288	11652665.5	48552.8	9.21
Total	448	126500672.0		

** Significant at *p* ≤ .01.

**TABLE 3:** Mean square from combined ANOVA for yield related traits of 25 cowpea landrace tested at six environments

Source	df	DF	DM	PH	PPP	SPP	HSW
Genotype	24	41**	100**	1135**	26**	6**	1103305**
Environment	5	1668**	3060**	98526**	5579**	203**	8681504**
Rep (Env.)	12	7ns	10ns	243**	109**	4**	261579**
Interaction	120	28**	74**	733**	42**	7**	652912**
Pooled error	288	4	10	88	5	2	48553

Abbreviations: DF, days to flowering; df, degrees of freedom; DM, days to maturity; HSW, hundred seed weight; ns, non‐significant; PH, plant height; PPP, pod per plant; SPP, seed per pod.

** Significant at *p* < .01.

* Significant at *p* < .05.

### 3.2 Additive main effects and multiplicative interaction (AMMI) analysis for grain yield

AMMI analysis for grain yield showed a highly significant (*p* ≤ .01) effect of environment, genotype, and genotype × environment interaction. The effects of environment, genotype, and genotype × environment interaction accounted for 29.79%, 15.60%, and 42.06% of the total sum of squares, respectively (Table 4). Most of the total sum of squares of the model was attributed to the interaction and environmental effects indicated larger differences in genotypic responses across environments and those environments were diverse. This also designated the reliability of the multi‐environment experiments. In harmony with this, Zali et al. (2012) in chickpea and Alemayehu et al. (2016) in groundnut had reported the large contribution of GEI than environment and genotype effects for the observed yield variation. The application of the AMMI model for partitioning the GEI (Table 4) revealed that the first four terms of AMMI were significant and explained 96.51% of the GEI. The first and second principal component axis (IPCA) of the interaction explained 34.26% and 28.88% of GEI sum of squares, respectively. AMMI model with first and second multiplicative terms is adequate for cross‐validation of the yield variation explained by GEI (Gauch et al., 2008).

**TABLE 4:** Additive main effect and multiplicative interaction analysis of variance for grain yield (kg ha^−1^) of cowpea genotypes across six environments

Source of variation	df	Sum of square	Mean square	Total variation explained (%)	G × E explained (%)
Treatments	149	152565439	1023929**	87.45	
Genotypes	24	27218792	1134116**	15.60	
Environments	5	51965751	10393150**	29.79	
Rep (Env.)	12	2756334	229694**	1.58	
Interactions	120	73380896	611507**	42.06	
IPCA 1	28	25142431	897944**		34.26
IPCA 2	26	21192008	815077**		28.88
IPCA 3	24	17637284	734887**		24.04
IPCA 4	22	6848898	311314**		9.33
G × E error	20	2560274	128014		3.49
Pooled error	288	19144635	66474	10.97	
Total	449	174466407	388567		

Note

Rep (Env.), replications within environment; G × E, genotype by environment interaction; IPCA 1 and IPCA 2, interaction principal component axis one and two, respectively.

** Significant at *p* ≤ .01.

Among the testing environments, grain yields were highest at Miesso as compared to the other five environments with a mean grain yield of 2528.8 kg ha^−1^ followed by Melkassa (2487.1 kg ha^−1^) and Kobo (1986.6 kg ha^−1^) (Table 5). The lowest grain yield was obtained at Sirinka with a mean yield of 1676.1 kg ha^−1^. The superior performance of genotypes at Miesso and Melkassa can be attributed to the uniform distribution of rainfall throughout the cropping season. The tested genotypes showed inconsistent yield advantage across environments. The mean grain yield of genotypes over environments in Table 5 indicated that G24 (2632 kg ha^−1^) and G16 (2290 kg ha^−1^) were the highest yielding genotypes whereas genotype G11 was the lowest yielder (1535 kg ha^−1^).

**TABLE 5:** Mean grain yield (kg ha^−1^) and environment and genotype IPCA1 scores for 25 genotypes tested at six environments during 2019

Code	Genotypes	Environments						Genotype	IPCA1
		Sekota	Kobo	Sirinka	Melkassa	Miesso	Babile	Mean	
G1	NLLP_CPC_07‐47	1829	1772	2590	2613	1777	2198	2130	−15.54
G2	NLL_CPC_07‐74	2109	2524	2362	2491	2658	1514	2276	0.98
G3	NLLP_CPC_07‐168	2002	1273	2536	2012	3070	1234	2021	−22.16
G4	NLLP_CPC_07‐27	2111	2393	1971	2370	3068	1589	2250	−0.96
G5	NLLP_CPC_07‐05	2085	2148	1702	2518	2952	1066	2078	0.81
G6	NLLP_CPC_07‐01	1838	1918	777	2602	2725	1441	1883	13.44
G7	NLL_CPC_07‐49	2043	1478	1831	2807	2690	2731	2263	−4.71
G8	NLLP_CPC_07‐77	1811	1410	2467	1925	2651	1406	1945	−20.24
G9	NLL_CPC_07‐46A	1839	1963	1223	2063	3198	1958	2041	5.67
G10	ACC 211557	1923	1652	1954	2468	2564	1716	2046	−7.53
G11	ACC222890	1434	1319	1679	2603	1123	1053	1535	−7.58
G12	Dass 002	1254	2184	1153	2021	1513	1657	1630	7.27
G13	Dass 007	1905	2667	1357	2171	2972	1249	2053	9.84
G14	NLLP_CPC_07‐69	1362	1732	1810	1866	1718	1395	1647	−7.92
G15	NLLP_CPC_07_	1853	1207	1357	2429	2792	1311	1825	−3.06
G16	ACC 233403	2098	2519	982	3058	2764	2322	2290	0.71
G17	NLLP_CPC_07‐28	1525	1908	787	2309	2216	1846	1765	12.12
G18	NLLP_CPC_07‐46B	1924	1566	1405	3410	1728	1942	1996	3.67
G19	ACC 244804	1598	2604	869	2597	1851	1507	1838	18.06
G20	NLLP_CPC_07‐29	2027	2002	1955	2261	3080	1953	2213	−4.47
G21	ACC 223402	2056	2314	1679	2664	2730	1748	2199	3.95
G22	NLLP_CPC_07_48B	1877	1670	2310	2434	2332	2343	2161	−12.43
G23	NLLP_CPC_07‐03	2108	1524	1196	2683	3365	2580	2242	5.25
G24	NLLP_CPC 07–55	2404	3049	2120	2861	3151	2208	2632	−3.09
G25	Kanketi	2190	2871	1830	2942	2531	1276	2273	5.75
Mean		1888	1987	1676	2487	2529	1730	2049.28	
Env. IPCA1		−0.75	27.64	−44.35	12.19	2.1	3.16		

Abbreviations: Env., environment; IPCA 1, interaction principal component axis.

The sign of the IPCA scores indicates the pattern of interaction of the genotypes across the environments and vice versa. Genotypes and environments with a similar sign of their IPCA sores interact positively for that trait. Sekota and Sirinka were different from the other locations in both the interaction and for the main effects (Table 5). These locations possessed a negative IPCA1 score and mean grain yield below the grand mean. while the other two locations Kobo and Babile possessed positive environment IPCA1 scores with mean grain yield below the grand mean whereas, Melkassa and Miesso possessed positive environment IPCA1 scores with mean grain yield above the grand mean.

By considering IPCA1 scores alone and regardless of the positive or negative signs, genotypes with large scores have high interactions (unstable), whereas varieties with small IPCA1 scores close to zero have small interactions and are stable (Zobel et al., 1988). Accordingly, genotypes: G16, G5, G4, G2, G15, and G24 showed relatively smaller IPCA1 scores thus are considered to be stable and had wider adaptation while, G3, G8, G19, and G1showed higher IPCA1 scores, respectively (Table 5). Similar to genotypes, environments with higher IPCA scores discriminate among genotypes more than environments with lesser scores. Thus, Sirinka was the most discriminating environment for the genotypes as indicated by the longest distance between its marker and the origin, followed by Kobo. However, due to their high IPCA scores, genotypic variability at this environment (Sirinka) may not exactly reflect the average performance across environments.

### 3.3 Four best genotypes selections of AMMI model

The highest yielding genotype (G24) was among the four best genotypes selected by the AMMI model and had selected as 1st best genotype at four environments and as 2nd best genotype at one environment (Table 6). This genotype was selected both at favorable environments (environmental mean yield greater than the grand mean) and unfavorable (environmental mean yield less than the grand mean), suggesting that it is desirable for cultivation in both environments. Similarly, the second‐highest yielder genotype (G16) was selected at two unfavorable environments and one favorable environment as 1st and 2nd best genotype whereas the third‐highest yielding genotype (G2) was selected at one unfavorable environment (sekota) as 3rd best genotype. According to AMMI's best four selections, Genotypes G24, G18, G4, and G25 were desirable for both favorable and unfavorable environments but G18 grain yield was lower than the grand mean. G9 and G13 were more desirable in favorable environments whereas G2, G1, G22, G8, G23, and G3 were desirable in unfavorable environments. The selection of these genotypes in respective environments by the AMMI model is an indication of the best adaptation of the genotypes at those particular environments.

**TABLE 6:** The first four best cowpea landraces selected for mean yield by the AMMI model per environment

Environment	Mean (kg ha^−1^)	IPCA score	1	2	3	4
Kobo	1987.0	27.64	G16	G24	G25	G23
Melkassa	2487.0	12.19	G24	G16	G18	G25
Babile	1730.0	3.16	G24	G16	G1	G18
Miesso	2529.0	2.1	G24	G4	G9	G13
Sekota	1888.0	−0.75	G24	G4	G2	G23
Sirinka	1676.0	−44.35	G1	G3	G8	G22
Grand mean	2049.5					

Abbreviations: kg ha^−1^, kilogram per hectare; IPCA, interaction principal component axis.

### 3.4 GGE biplot for evaluation of genotypes and environments

**FIGURE 1:**
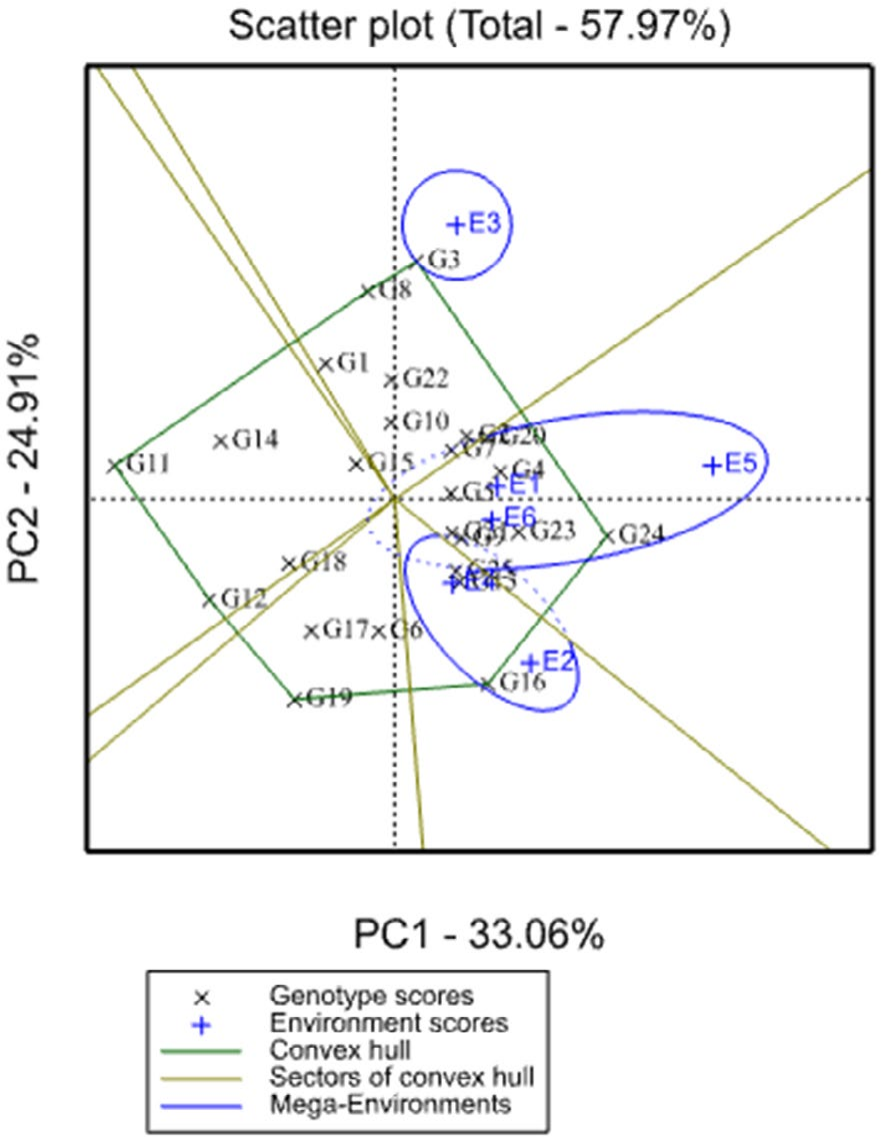
Polygon view of genotype by environment interaction for cowpea genotypes

The residual mean square from the AMMI model for grain yield was highly significant (Table 4) which suggested that the importance of constructing GGE biplot to visualize “Which‐won‐where” Patterns of genotypes and environments and the discriminating ability and representativeness of the environments. In the present study, the GGE biplot graphic analysis of 25 cowpea genotypes revealed that the first two principal components explained 57.97% of the total GEI variance (Figure 1).

A polygon view of the GGE biplot was formed by connecting the vertex genotypes with straight lines and the rest of the genotypes were placed within the polygon. G3, G24, G16, G19, and G11 were vertex genotypes and they are best in the environment lying within their respective sector in the polygon view of the GGE biplot (Mehari et al., 2015); thus these genotypes performed either the best or the poorest in one or more locations since they had the longest distance from the origin of the biplot. According to Yan and Tinke (2006) and Gauch et al. (2008), genotypes within the polygon and nearer to the origin of the axes have wider adaptation and less response for environmental variation. Yan and Rajcan (2002) reported that responsive genotypes were those having either best or the poorest performance in one or all environments. G24 and G16 were identified as the highest yielding genotypes whereas G19 were considered as the lowest yielding genotype among vertex genotypes. In addition, no environment fell inside the sectors of the vertex genotypes G11 and G19, which indicated that those vertex genotypes were not the best in any of the test environments.

Another interesting feature of the GGE biplot is the identification of mega‐environments as well as their winning genotypes. The present investigation suggested the existence of three cowpea growing mega‐environments (ME1, ME2, and ME3) in Ethiopia as shown in Figure 1. Among the testing environments, Sekota (E1), Miesso (E5), and Babile (E6) fell inside mega‐environment one (ME1), Sirinka fell inside mega‐environment two (ME2) whereas Kobo (E2) and Melkassa (E4) fell inside mega‐environment three (ME3). The vertex genotypes in each sector are the best genotype in environments whose markers fall into the respective sector. Environments within the same sector share the same winning genotypes, and environments in different sectors have different winning genotypes. Accordingly, Genotypes G24, G3, and G16 are suggested as the winner and highest yielding genotypes in mega‐environment one, two, and three, respectively. Yan et al. (2002) reported that the polygon view of GGE biplot is the best way for the identification of winning genotypes with visualizing the interaction patterns between genotypes and environments.

**FIGURE 2:**
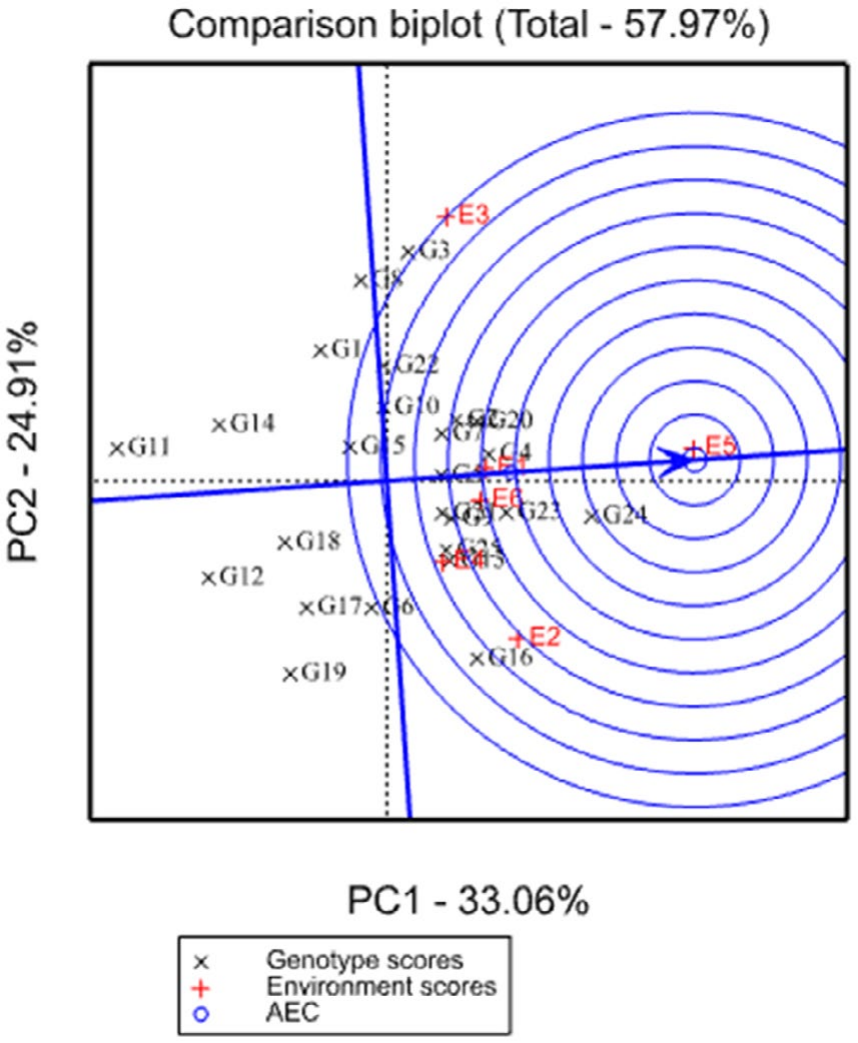
GGE‐biplot based on the ranking of genotypes for grain yield relative to an ideal genotype

An ideal genotype is defined as a genotype with the greatest PC1 score (mean performance) and with zero GEI, as represented by an arrow pointing to it (Figure 2). Even though such type of genotype may not exist in reality, it can be used as a reference for the evaluation of genotypes (Yan, 2002; Yan & Tinke, 2006). If a genotype is located closer to the ideal genotype, it becomes more desirable than other genotypes which are located far away from the ideal genotype. Therefore, concentric circles were drawn around the central circle which contains the ideal genotype to visualize the distance between each genotype and the ideal genotype. From the present investigation, G24 was the “ideal” genotype, with the highest mean grain yield and thus considered as the most stable across variable environments. Simultaneously, G23, G4, and G20 genotypes were located closer to the ideal genotype and were considered as desirable genotypes.

#### 3.4.1 Discriminating ability and representativeness of environments

**FIGURE 3:**
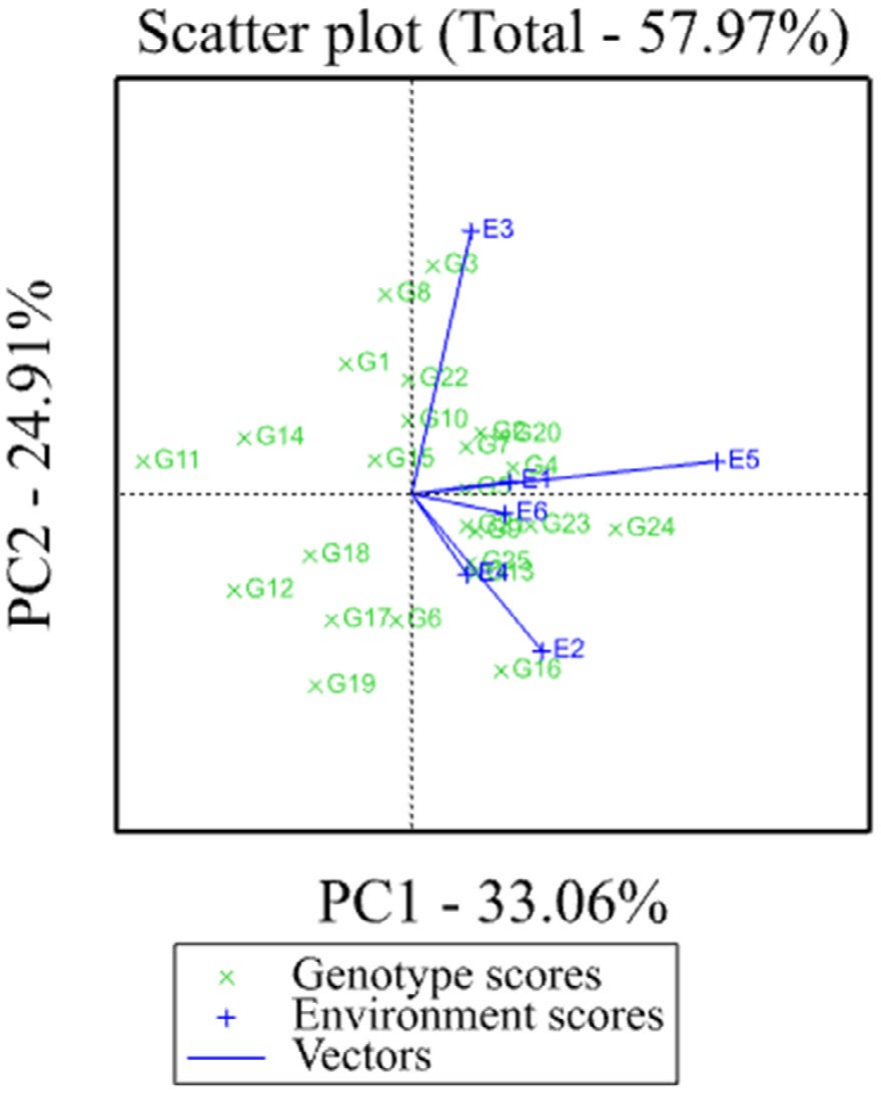
Discriminating power and representativeness of test environments

According to Yan et al. (2002), the discriminating ability and representativeness view of the GGE biplot is the important measure of test environments, which provide valuable and unbiased information about the tested genotypes. Yan and Tinke (2006) also reported that Environments with longer vectors had the more discriminating ability of the genotypes whereas environments with very short vectors had little or no information on the genotype difference. From this study, the test environments Sirinka (E3) and Miesso (E5) were identified as the most discriminating environments which provided much information about differences among genotypes, while Sekota (E1), Melkassa (E4), and Babile (E6) provided little information about the genotype differences (Figure 3).

Another equally important measure of a test environment is its representativeness of the target environments. If a test environment is not representative of the target environments, it is not only useless but also misleading since it may provide biased information about the tested genotypes (Yan & Kang, 2003). To know the representativeness of a test environment, understanding some important terms such as average environment (the small circle used as a benchmark for measuring the representativeness of a test environment), the average environment coordinate axis (the line that passes through the biplot origin and the average environment), and environmental vector (the line that connects the origin of biplot and a testing environment) is a very crucial task before measuring the representativeness of a test environment. Thus, based on the size of the angle between the vector of an environment and the abscissa of the average environment coordination (AEC) axis, it is possible to measure the representativeness of a testing environment. That is, a testing environment that makes an acute angle with AEC axis has a positive correlation with other testing environments and it is considered as a representative of the other testing environments, whereas the testing environment that makes an obtuse angle with AEC axis has a negative correlation with other testing environments and least representative (Yan et al., 2007; Yan & Tinke, 2006). From this study, Miesso was identified as the most representative testing environment, which was able to provide unbiased information about the performance of the tested genotypes, whereas Sirinka was identified as the least representative testing environment (Figure 3).

**FIGURE 4:**
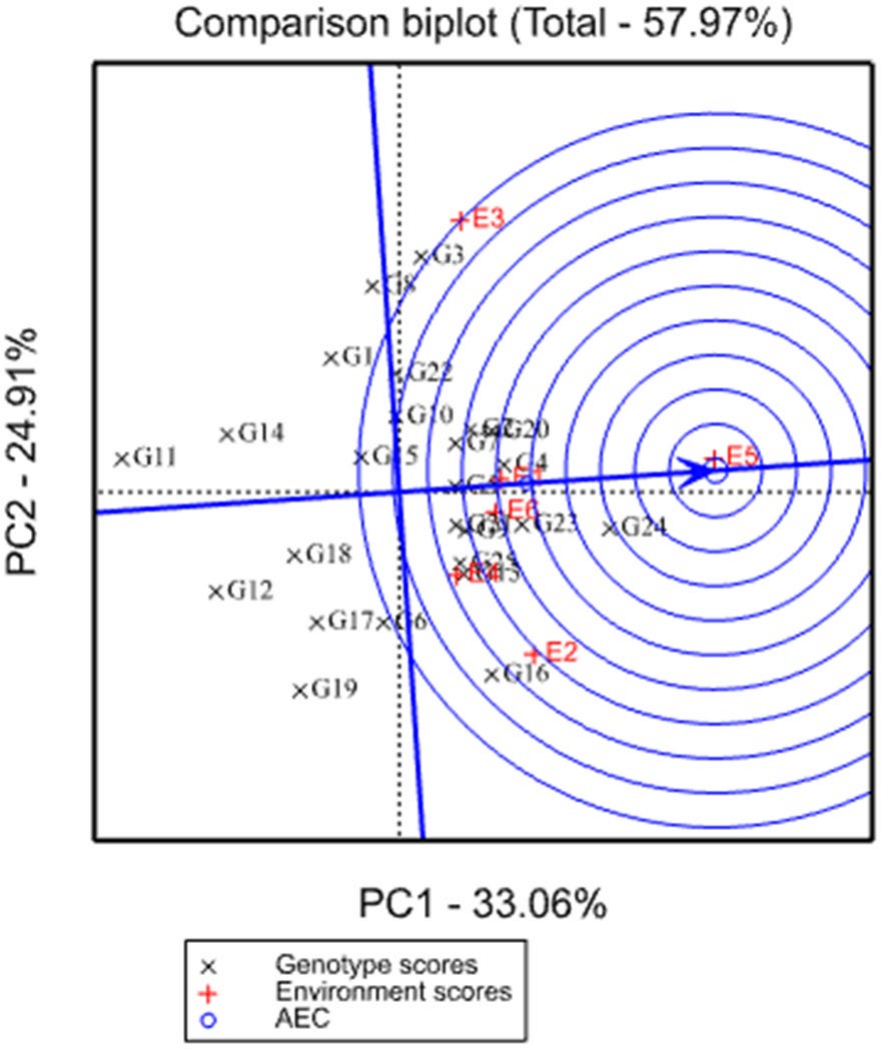
GGE‐biplot based on environment‐focused scaling for comparison of the environments with the ideal environment

The ideal test environment is an environment that has more power to discriminate genotypes in terms of the genotypic main effect as well as being able to represent the overall environments. It is used for selecting generally adaptable genotypes but obtaining such type of environment is very difficult in real conditions. In such condition, environments that fell near to a small circle located in the center of concentric circles and an arrow pointing on it (ideal environment) is identified as the best desirable testing environments (Yan & Rajcan, 2002). Among the testing environments used in this study, Miesso (E5) was identified as an ideal environment in terms of being the most representative of the overall environments and powerful to discriminate genotypes (Figure 4). Discriminating but non‐representative test environments like Sirinka (E3) are useful for selecting specifically adaptable genotypes if the target environments can be divided into mega‐environments or it is useful for culling unstable genotypes if the target environment is a single mega‐environment.

## 4 CONCLUSION AND RECOMMENDATION

Combined analysis of variance over six locations showed significant differences among genotypes, environments, and genotypes × environments interaction (GEI) for grain yield and most of the yield‐related traits. The significant genotypes × environments interaction effects indicated the inconsistent performance of genotypes across the tested environments and the differential discriminating ability of the tested environments.

Among the tested environments, the highest mean grain yield (2529 kg ha^−1^) was registered at Miesso followed by Melkassa (2487 kg ha^−1^) while Sirinka (1676 kg ha^−1^) and Babile (1730 kg ha^−1^) were the least yielding environments. The highest grain yields were obtained from G24 (2632.4 kg ha^−1^), G16 (2290.4 kg ha^−1^) and G2 (2276.3 kg ha^−1^) while the lowest grain yield was obtained from G11 (1535.0 kg ha^−1^) genotype. The significance of GEI suggested the need to conduct further analysis on GEI to understand the nature of the interaction, and to identifying stable genotypes.

AMMI analysis revealed a highly significant (*p* ≤ .01) effect of environment, genotype, and their interaction on grain yield. The effects of environment, genotype, and interaction accounted for 29.79%, 15.60%, and 42.06% of the total sum of squares, respectively. The first four terms of AMMI were significant and explained 96.51% of the GEI. The first and second principal component axis (IPCA) of the interaction explained 34.26% and 28.88% of GEI sum of squares respectively. AMMI model selected G24 as 1st best genotype at four environments and as 2nd best genotype at one environment. This genotype was selected both at favorable and unfavorable environments, suggesting that it is desirable for cultivation in both environments.

The polygon view of the GGE biplot identified three mega‐environments (ME1, ME2, and ME3) with winning genotypes: G24, G3, and G16 respectively. The highest productive (2528.8 kg ha^−1^) environment, miesso has been identified as the most; discriminating and representative testing environment whereas the lowest productive (1676.1 kg ha^−1^) Sirinka was the least discriminating and representative. The highest yielder genotype G24 (2632 kg ha^−1^) was identified as the “ideal” and the most stable genotype followed by G2 (2276 kg ha^−1^), G4 (2250 kg ha^−1^), G20 (2213 kg ha^−1^) and G16 (2290 kg ha^−1^) were most stable genotypes with no statistical significant difference in mean grain yield, however, only the first three genotypes exceeded the standard check variety kanketi in grain yield. Therefore, genotypes G24 and G16 were recommended for verification and commercial production in most cowpea growing areas of Ethiopia.

## CONFLICT OF INTEREST

The authors declared no conflict of interest.

## Data Availability

The data that support the findings of this study are openly available in Yirga at https://www.researchgate.net/profile/Yirga_Wasihun.
